# Mutation detection in the non-coding genome

**DOI:** 10.1515/medgen-2021-2070

**Published:** 2021-08-14

**Authors:** Markus Schuelke

**Affiliations:** Klinik für Pädiatrie mit Schwerpunkt Neurologie, Charité – Universitätsmedizin Berlin, Berlin, Germany

It is indisputable that the field of medical genetics has tremendously profited from the advent of massive parallel sequencing technologies, i. e., next-generation sequencing (NGS). These technologies led to a surge of new disease gene discoveries and revolutionized our approach to the molecular diagnosis of patients with suspected rare genetic disease. Though NGS has become an integral part of routine clinical diagnostics and patient management for this patient population, the molecular diagnosis rate for monogenic disorders has leveled off at a maximum of ≈50 %. Physicians and researchers are therefore well aware that the competitive pricing of whole genome sequencing does not automatically translate into increased rates of molecular diagnosis for this patient group.

The vast majority of disease-causing genetic alterations known to date impact the coding sequences of proteins, thereby affecting protein structure, protein interactions, and protein function. This information is encoded in the exome, i. e., the transcribed part of the genome, which constitutes less than 2 % of the human genome. Our understanding of the rules that govern gene transcription and translation enables us to interpret and predict the effect of genetic variants in protein-coding regions. This stands in stark contrast to our limited ability to correctly interpret the vast amount of regulatory information encoded in the 98 % of the genetic material located outside of the coding regions. Gene regulation exerts multiple interwoven layers of control that when correctly tuned result in expression of the right proteins at the right time, place, and concentration. Any perturbance of this finely tuned system can lead to disease. Our DFG-funded research unit FOR2841 “*Beyond the Exome*” works on how our understanding of the non-coding genome can be improved and how we can translate this knowledge into an improved molecular diagnosis rate for patients with suspected rare genetic disease.

In this issue, we highlight the problems that have to be solved and the current methods for discovering and interpreting disease-causing variants in the non-coding genome. Krude et al. discuss several examples of non-coding genetic variants that disrupt gene function and lead to disease. Known disease-causing variants may affect splicing, impact the stability of triplet repeats, alter promoter and enhancer function, or, in case of structural variants, disrupt the entire 3D genomic structure. Because gene regulation is highly cell-, tissue-, and organ-specific, it is essential that we explore the regulatory features for each developmental step and disease group separately. We have selected examples from the three disease groups that form the clinical research focus of our research unit.

It is self-evident that non-coding variants have to be identified before we can interpret them. Larger structural variants are likely to be missed if we only employ standard short-read Illumina sequencing techniques. This is especially true for larger repetitive regions and for complex structural variants with rearranged or inverted genetic material. Schwarz et al. describe novel sequencing methods, such as linked-read and long-read genome sequencing, that aim to reconstruct complete alleles. This provides phase information with the ultimate goal of reading chromosomes from telomere to telomere. The article describes available and novel bioinformatic tools that help annotate and evaluate these genomic variants with respect to their disease-causing potential, including an inventory of existing tools and projecting future development. Access to effective, user-friendly bioinformatic tools is a necessity for the successful interpretation of non-coding variants in the context of disease phenotypes.

Transcription factors (TFs) are integral to gene regulation. These proteins enhance or inhibit the assembly of the transcriptome complex by interacting with specific non-coding DNA sequences in enhancer and promoter regions of their target genes. We currently lack precise prediction algorithms for TF binding sites (TFBSs), making it impossible to predict the effect of a mutation in a TFBS with confidence. This severely impedes our ability to annotate non-coding variants. Leiz et al. describe a set of biochemical, biophysical, and crystallographic methods that evaluate TF binding, both in specific experiments that assess a single TFBS variant and in those that generate a bird’s eye view of the binding characteristics of large sets of TFs. Novel artificial intelligence techniques have the potential to predict TF binding in the future. The ability to formulate rules for TF:DNA interaction will substantially improve our ability to interpret non-coding variants in a disease context.

In addition to TFBSs, many other regulatory features influence human gene transcription. These comprise promoters and enhancers, epigenetic modifications, e. g., histone modifications and methylation of CpG islands, regulation of chromatin accessibility, and the overall 3D genome structure. Guo et al. describe novel experimental techniques used to identify such regulatory features. As epigenetic modifications are highly dynamic, they have to be studied at different developmental stages and functional tissue states. The article describes the concept and experimental study of topologically associating domains (TADs), which comprise functional genomic modules where distal-proximal regulatory interactions take place. Disruption of TAD boundaries by mutations can result in complex developmental phenotypes. A detailed understanding of the multitude of layers in the regulation of the genome will improve the molecular diagnosis rate on a significant scale.

The current knowledge of gene regulation is highly fragmented and a substantial amount of information is not readily accessible to computer-based analysis software. Only a small percentage of information is stored in structured and curated databases that allow for bioinformatic analysis of regulatory variants in whole genome datasets from patients. The problem is further compounded by the lack of a uniform nomenclature for gene names and regulatory elements, as well as by the multitude of different methods that have been used to study gene regulation. Garda et al. describe current information sources, i. e., databases and repositories, as well as strategies for extracting computer-readable knowledge about regulatory features of the genome from the substantial body of published scientific literature. This process of “information extraction” will undoubtedly help connect a specific genetic regulatory variant to a patient phenotype.

Researchers and clinicians know that a substantial amount of work still remains in fields such as genetics, molecular biology, biochemistry, biophysics, and (bio)informatics before we will be able to confidently interpret whole genome datasets at a level comparable to how we currently interpret whole exome datasets. In order to reach this goal, we need to translate technological advances in sequencing technologies and related fields into progressively granular information on gene regulation down to the single-cell level as well as up to the systems level. Only then will we be able to close in on the overarching goal of FOR2841 “*Beyond the Exome*”: To end the diagnostic odyssey for all patients with suspected rare genetic disease.

